# Effects of host species and environmental factors on the prevalence of *Batrachochytrium dendrobatidis* in northern Europe

**DOI:** 10.1371/journal.pone.0199852

**Published:** 2018-10-25

**Authors:** Simon Kärvemo, Sara Meurling, David Berger, Jacob Höglund, Anssi Laurila

**Affiliations:** Department of Ecology and Genetics/Animal Ecology, Uppsala University, Uppsala, Sweden; Imperial College Faculty of Medicine, UNITED KINGDOM

## Abstract

The fungal pathogen *Batrachochytrium dendrobatidis* (*Bd*) poses a major threat to amphibian populations. To assist efforts to address such threats, we examined differences in *Bd* host infection prevalence among amphibian species and its relations to both local environmental factors in breeding habitats and landscape variables measured at three scales (500, 2000 and 5000 m radii) around breeding sites in southernmost Sweden. We sampled 947 anurans of six species in 31 ponds and assessed their infection status. We then examined correlations of infection prevalence with canopy cover, pond perimeter and pH (treated as local-scale pond characteristics), and the number of ponds, area of arable land, area of mature forest, number of resident people and presence of sea within the three radii (treated as landscape variables). The *Bd* infection prevalence was very low, 0.5–1.0%, in two of the six anuran species (*Bufo bufo* and *Rana temporaria*), and substantially higher (13–64%) in the other four (*Bombina bombina*, *Bufotes variabilis*, *Epidalea calamita*, *Rana arvalis*). In the latter four species *Bd* infection prevalence was positively associated with ponds’ pH (site range: 5.3–8.1), and negatively associated with areas of mature forest and/or wetlands in the surroundings. Our results show that the infection dynamics of *Bd* are complex and associated with host species, local pond characteristics and several landscape variables at larger spatial scales. Knowledge of environmental factors associated with *Bd* infections and differences in species’ susceptibility may help to counter further spread of the disease and guide conservation action plans, especially for the most threatened species.

## Introduction

Population and infection dynamics of pathogens that infect multiple host species are likely to differ in several ways from those of pathogens restricted to a single host [1]. Multi-host pathogens are generally better invaders, and have both more diverse arrays of susceptible hosts and higher rates of transmission between host species [2]. Thus, multi-host pathogens may pose higher risks of infection for rare host species (and severe decline or even extinction for endangered species) than single-host pathogens. However, the prevalence of infection often varies substantially among host taxa [3, 4]. Environmental factors can also influence pathogen dynamics, but their effects are often poorly understood and may be scale-dependent [5, 6]. At local spatial scales, the occurrence of infections may be associated with factors including habitat quality [7], host population density [8], host migration [9] and interactions among host species [10]. At a regional scale, the risk of infections may also be associated with factors such as landscape characteristics and climatic conditions [11, 12]. Anthropogenic impacts due to agricultural intensification, urbanization, and transport of pathogenic agents can also affect infection risks at different spatial scales [5, 13]. Thus, identifying combinations of species, habitats and environmental factors associated with high infection risks may be crucial for mitigating diseases [3, 4], and sound understanding of multi-host pathogens’ population and infection dynamics may be essential for formulating effective conservation programs.

Amphibians are among the most threatened and severely declining vertebrates globally [14, 15]. Thus, robust conservation actions are urgently needed for many amphibian taxa. Several multi-host pathogens affect amphibians [16] and one of the most serious threats to amphibian populations is the chytrid fungus *Batrachochytrium dendrobatidis* (*Bd*), which has been detected in ca. 700 amphibian species [17], and caused mass mortality and population declines all over the world [14–19]. *Bd* infection can result in chytridiomycosis, a disease affecting amphibians’ epidermis and outer keratinized layers, disrupting the transport of water, oxygen and salts. *Bd* infection dynamics are complex and depend on host species, genotypes and phenotypes of the fungus, and environmental factors [17, 20]. Consequently, *Bd* infections do not occur uniformly in the landscape. Instead, ponds and wetlands hosting amphibians typically differ in infection status [21–26]. Moreover, variations in *Bd* infection prevalence among amphibian species have been observed, and attributed to variations in, for example, temperatures in breeding habitats [27], environmental conditions more generally [21, 26], immune responses [28], and skin microbial communities [29]. However, although chytridiomycosis has been called “the worst infectious disease ever recorded among vertebrates in terms of the number of species impacted, and its propensity to drive them to extinction” [30], there are significant gaps in knowledge of differences in resistance among amphibian species and dispersal of *Bd* across landscapes.

More knowledge of the factors associated with occurrence of the fungus at different spatial scales in the landscape would greatly facilitate efforts to understand the variations in *Bd* infection prevalence among taxa and habitats. While temperature and precipitation appear to be important environmental factors associated with the occurrence of *Bd* infections at large geographical scales [17, 31–34], factors affecting *Bd* at smaller scales seem to be more multifactorial and ambiguous. Several studies have shown that *Bd* occurrence may be associated with habitats’ microclimates (e.g. [25, 27, 35, 36]). In tropical regions, higher infection prevalence has been recorded among amphibians in cool forest ponds and forest-dwelling amphibian individuals than among those in warmer open-canopy ponds or open areas ([4, 36, 37], but see [38]). Dry conditions may reportedly reduce survival of *Bd* [39], and several authors have observed higher infection prevalence among amphibians in large permanent ponds and streams than among those in temporary ponds with higher desiccation risks ([21, 37, 38, 40] but see [20]). Similarly, several authors have found indications that numbers of surrounding ponds may be positively related to infection risks [37, 41].

In Europe, increases in agricultural areas in the surrounding landscape reportedly increase infection risks [40], and in both subtropical and temperate zones the extent of surrounding forest and amphibian species richness are positively associated with risks of *Bd* infection [22, 24, 31, 37]. Urbanization and human activities may also be positively associated with *Bd* infection risks, as humans may directly or indirectly act as vectors, or because high environmental stress due to interacting factors such as pollution may affect the host immune system ([24, 40, 42] but see [43–44]).

Few studies have investigated factors affecting *Bd* infection risks in cool temperate or boreal regions with relatively low summer temperatures (but see [45–48]) where climate and, consequently, disease dynamics differ from those in warmer regions. In Sweden, *Bd* was first discovered in 2010 [49], followed by records on several amphibian species in southernmost Sweden and the Stockholm area, providing the northernmost records of *Bd* in Europe (S. Meurling et al., in prep.). However, no *Bd* was found in an examination of 197 Swedish samples of museum specimens collected between 1994 and 2004 [50], raising the possibility that *Bd* has colonized Sweden relatively recently. Currently, the environmental factors affecting the occurrence of *Bd* in northern Europe and at higher latitudes remain largely unexplored [40].

Thus, the aim of this study was to study *Bd* infection prevalence across an array of host species and its associations with pond characteristics and landscape factors in northern Europe. To do so, we sampled adult amphibians from breeding aggregations in 31 wetlands in southernmost Sweden, then assessed their infection status and its relations to selected environmental variables. We expected to find inter-specific variations in *Bd* prevalence among amphibian species, with pronounced infection risks for rare species [2]. We also expected infection prevalence to be: correlated with pH, due to its effects on *Bd* growth [51]; positively associated with pond perimeter and canopy cover [4, 40]; and associated with landscape-scale factors through their effects on dispersal ability and potential vectors’ habitat choice [52]. We analysed the prevalence (proportion of infected individuals in each pond) of *Bd* infection while controlling for effects of different species in the models. We specifically addressed the following four questions: How does *Bd* infection prevalence vary among different host species? Which environmental factors influence the prevalence of *Bd* infection in these species? Are local pond characteristics or landscape-scale factors more important predictors of *Bd* infection prevalence? At which spatial scales are the environmental factors most influential?

## Materials and methods

**Fig 1:**
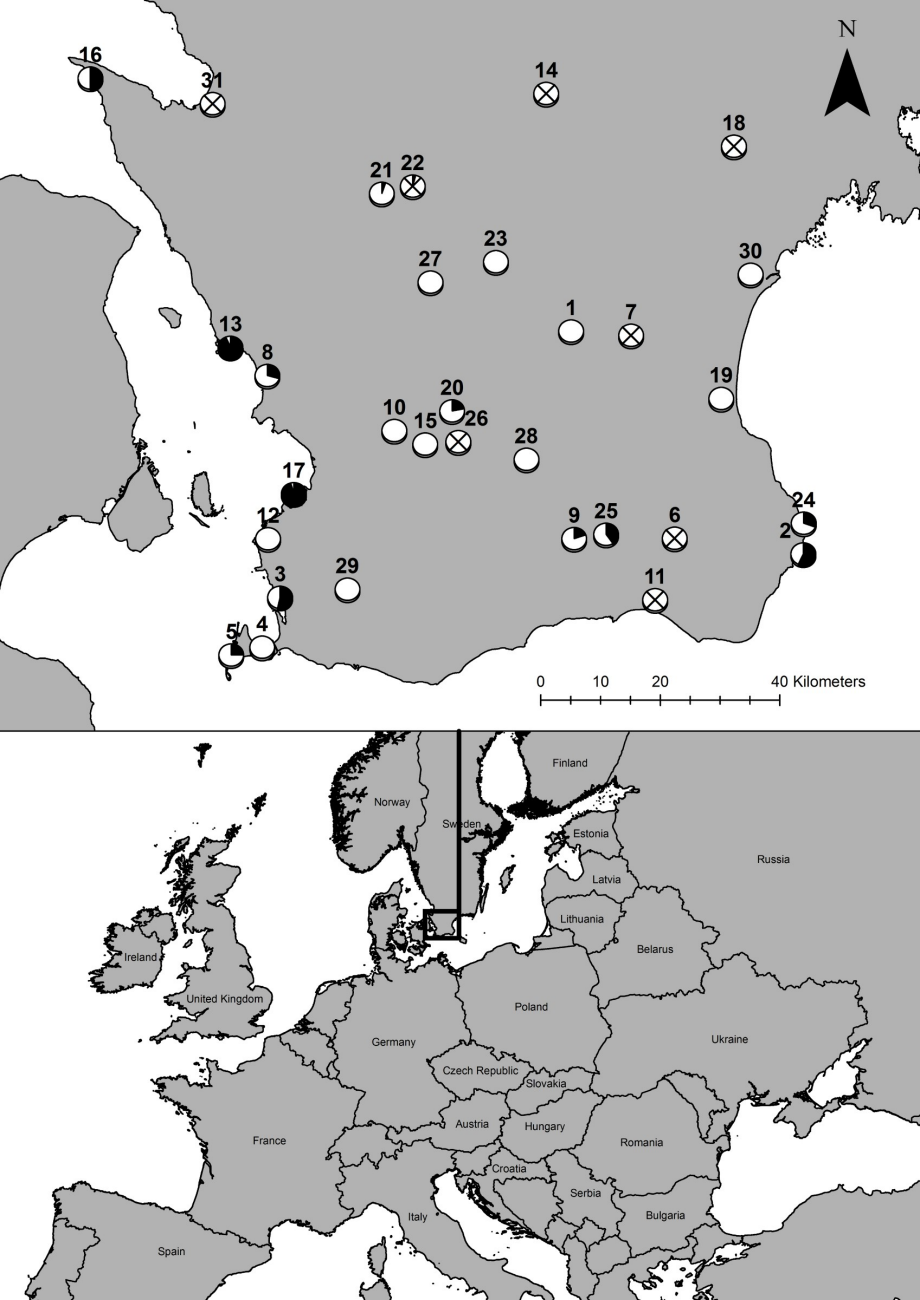
Site map. Location and prevalence of *Bd* infection in amphibians (black indicates the proportion of recorded individuals with *Bd*) in the 31 studied ponds in southern Sweden. Only *Bufo bufo* and *Rana temporaria* (with very low prevalence of *Bd* infection) were found at sites marked with X. They were not included in the local and landscape models.

We sampled anurans at 31 ponds spread across southern Sweden (Scania province) in March-May 2015 and April 2016 (Fig 1 and S1 Table: including site coordinates). This region is mainly dominated by arable lands with scattered mixed woodlands. The study ponds were selected based on earlier knowledge of breeding habitats and by visual inspection of maps indicated that they were likely breeding sites. Twenty-three ponds were sampled in 2015 and thirteen in 2016, five ponds in both years. Following the exclusion of two species from the local- and landscape-level modelling (see Results), samples from 20 ponds were used in 2015 and five in 2016 (two of which were sampled in both years). Each pond was only used once to control for site replication in the models (see Statistical analyses). We screened for *Bd* infection by capturing adults from breeding aggregations by hand or with nets, then used a standard swabbing protocol with 25 strokes per individual [53]. The swabs were preserved in alcohol in 2015 and in Dryswab (MWE MW110) in 2016 before analysis.

### Study species

Ten species of anurans are found in Scania and six of these species were sampled in this study. Two of the sampled species, the common toad *Bufo bufo* and common frog *Rana temporaria* are common and widespread across Sweden, including Scania. The remaining four species (the moor frog *R*. *arvalis*, fire-bellied toad *Bombina bombina*, green toad *Bufotes variabilis*, and natterjack toad *Epidalea calamita*) are all protected under the European Habitats Directive [54]. Of these, *R*. *arvalis* is a common and widespread species whereas *B*. *bombina*, *B*. *variabilis* and *E*. *calamita* are uncommon and local species in Sweden, and their distributions are limited to the southern part of the country. *Bufotes variablis* and *E*. *calamita* are listed as vulnerable (VU) in the Swedish Red List, *Bombina bombina* was removed from the list in 2015, and *R*. *arvalis* has not been included in the list [55].

### Molecular analyses

DNA was extracted from swabs using a Qiagen DNeasy Blood and Tissue kit (Qiagen, ref. 69506) following the standard protocol with modifications suggested by [56]. Presence of *Bd* was assessed by qPCR amplification of the internal transcribed spacer (ITS)-5.8S rRNA region [57], in 25 μl reaction mixtures containing 12.5 μl of 2X Taqman Master Mix (Applied Biosystems, ref. 4318157), 2.25 μl of 10 μM solutions of the forward and reverse primers, 0.625 μl of 10 μM MGB probe and 5 μl of DNA solution (diluted x10 in water). Each sample was assayed in triplicate. To detect possible false negatives caused by inhibitors, to one of each set of triplicates we added an exogenous internal positive control (IPC; [57]), consisting of 1 μl of 10X Exo IPC Master Mix and 0.5 μl of 50X Exo IPC DNA (VICTIM dye, Applied Biosystems ref. 4304662).

We used a Biorad CFX96 system and previously described amplification conditions [57], with standards of 0.1, 1, 10 and 100 genomic equivalents (GE) (2016 samples) or a positive control of approximately 1–10 GE (2015 samples), for the qPCR assays. An individual was scored as *Bd*-positive if one or more of the triplicate samples exhibited a positive signal (i.e. a clear exponential amplification curve). If the IPC showed signs of inhibition (no curve), negative samples were re-run once before being designated unscoreable.

### Local variables

Three local habitat characters of each pond were evaluated: Perimeter, pH and Southern canopy cover. Pond perimeter is highly correlated with pond size and chosen for use in the model as vegetated shallow shores are important breeding aggregation sites of amphibians [58]. Perimeter values for sufficiently large ponds were available from topographic maps (Swedish National Land Survey) using ArcMap (ArcGIS, ESRI, Redlands, CA, USA). These ponds are large and size do not vary considerably over time, relatively to smaller pond sizes [59]. Perimeter of smaller ponds not visible from satellite maps were obtained from polygons drawn in Google Earth and transformed to perimeter in Earth Point (www.earthpoint.us/Shapes.aspx). As small ponds temporally may change in size, screening dates were chosen to correspond to the actual pond sizes from the field. Values of pH and Southern canopy cover (canopy cover along the southern shore; hereafter Canopy) were obtained in October 2017. Ponds with more canopy cover have lower and less variable water temperatures than open-canopy ponds [60] and southern canopy was chosen as this is the main contributor to shading of the ponds. The pH values were determined by single measurements with a ThermoFisher Orion 131S pH-meter and Canopy was estimated visually by the same person in 10% categories, following a previously described rationale [60]. Recordings in October were considered to correspond to general values, as there are small seasonal variations in pond pH [61, 62] and this period is the onset of the defoliation period of deciduous trees in southern Sweden.

### Landscape variables

Seven characteristics of surrounding wetlands, forests, arable land and urban features were used as landscape variables in the modelling. We examined correlations between all local and landscape variables, and only retained those with a correlation coefficient <0.7 in the models to avoid co-linearity. Consequently, we excluded three landscape variables (total perimeter of surrounding ponds, total length of roads and marshland area) from the seven initial variables. The four remaining variables—number of surrounding ponds (hereafter Surrounding ponds), area of arable lands (crops and fruit farms, hereafter Arable lands), area of mature forest (timber volume >300 m^3^ha^-1^, hereafter Mature forest), and number of resident people (hereafter Resident people)—were determined in three nested circular buffer zones with widths of 500, 2000 and 5000 m around the perimeter of the focal ponds, using the function “spatial join” in ArcMap. The buffer sizes were based on general maximum movement distances of amphibians [63]. An additional binary variable—presence of sea (hereafter Sea)—was included in the models to account for its negative relationship with land area.

Values of the variables Surrounding ponds, Arable lands, and Sea were extracted from topographic vector maps published by the Swedish National Land Survey. Arable lands were converted to raster format and quantified with 10 x 10 m resolution. Mature forest was quantified using kNN-raster data obtained from the Swedish Forest Agency [64] originally at 25 x 25 m resolution and aggregated to 50 x 50 m by averaging, due to low volume accuracy at the original scale [65]. Resident people was summed from a geographical point-layer dataset created by the Swedish Bureau of Statistics. Differences in buffer size around ponds with different sizes of edges could have had confounding effects, particularly as some of the ponds were very small. Therefore, all landscape variables were corrected by a factor calculated by dividing each buffer area (a full circle with a radius corresponding to the buffer zone width) by the smallest buffer size at each landscape scale [66]. The landscape data were processed in ArcMap (ArcGIS 10.4, ESRI, Redlands, CA, USA). All continuous variables were standardized to a mean of zero using the scale function in R [67].

### Statistical analyses

Effects of three pond and five landscape variables as well as species identity were analysed with *Bd* infection prevalence as the binomially distributed response variable in mixed effects models with *Site* specified as a random effect to attain the correct level of replication for the fixed effects. Effects of the pond variables were analyzed using one model and effects of the landscape variables within the three distances from the ponds (500, 2000 and 5000 m) using three separate models (Table 1) to avoid potential obscuration of important effects of landscape-scale factors [68]. Due to non-convergence in some of the models using traditional maximum likelihood methodology, we ran Bayesian linear mixed effect models with Markov-chain Monte Carlo simulations available in the package MCMCglmm [69]. We used variance expanded priors for the random effects to improve sampling properties of the MCMC chains [70]. We ran the models with residual variance fixed to 1 (as standard for binomial response models) and a flat prior on the probability scale for the fixed effects. This is a recommended procedure when the number of observations in some cells are low [70] (as is the case for *Bd* infection prevalence at some sites and in some species) and the data show near complete separation [71]. Models ran for 2,100,000 iterations, preceded by 100,000 discarded “burn-in” iterations. We saved every 2000th iteration, resulting in 1000 stored and uncorrelated (all autocorrelations <0.05) posterior estimates of model parameters upon which we based our Bayesian p-values and 95% credible intervals. While we focus on the results from these more robust Bayesian models, we note that the (converged) maximum likelihood analyses gave similar qualitative results.

**Table 1:** Variables included in the four main models of effects of environmental factors used in the study.

Effects	Local factors	Landscape, 500 m	Landscape, 2000 m	Landscape, 5000 m
Random	Site	Site	Site	Site
Fixed	Species	Species	Species	Species
	pH	Forest	Forest	Forest
	Perimeter	Arable land	Arable land	Arable land
	Canopy	Surrounding ponds	Surrounding ponds	Surrounding ponds
		People	People	People
		Sea	Sea	Sea

Site and Species refer to the studied ponds and amphibian species. The local factors pH, Perimeter and Canopy respectively refer to the ponds’ pH, circumference, and percentage cover by the southern canopy. Forest, Arable land, Surrounding ponds and People respectively refer to the landscape factors area of mature forest (>300 m^3^ timber ha^-1^), area of crops and orchards, number of surrounding ponds and number of resident people in the surrounding landscape (within 500, 2000 and 5000 m of the ponds).

To test for differences in *Bd* infection prevalence among species, we first ran an initial model including all six sampled amphibian species, with *Site* specified as a random effect, but excluding the environmental factors. To test effects of the environmental factors we ran a model excluding the common species (*Bufo bufo* and *R*. *temporaria*) with near zero *Bd*-prevalence, due to the absence of incidence and variation of infection in them. The four remaining species (fixed effect) and Site (random effect) were included in all models. The set of explanatory variables (fixed effects) used in a model examining effects of local environmental factors were Perimeter, pH and Canopy (Table 1). The set of explanatory variables (fixed effects) for estimating the influence of the landscape factors were Surrounding pond, Sea, Resident people, Mature forest and Arable land. The three nested distance scales (500, 2000, 5000 m) were not independent of each other, and separate analyses were conducted at each scale with the same response variable. Consequently, four main analyses were conducted (Table 1).

We compared model weights of the MCMC models with the function model.sel from the MuMIn package implemented in R [72] to estimate effects of local and landscape-scale variables.

### Ethics statement

All methods were carried out in accordance with relevant guidelines and regulations and all experimental protocols followed Uppsala University guidelines. Sampling procedures of all amphibian species in this study were approved by the Ethical Committee for Animal Experiments in Uppsala County (C 28/15) and collection permits of the common and protected amphibian species were provided by the Scania County Board. Land owners gave permission to conduct the study on their lands. No further permissions were needed.

## Results

Overall, *Bd* infected anurans were detected in 13 of the 31 surveyed ponds (42%), and 156 of the total 947 individuals were infected (16%). At least one infected individual of each sampled species was infected, but *Bd*-infection prevalence differed strongly among the species (0.5–64%; Table 2) and sites (0–97%; S1 Table). The detected *Bd* infection prevalence was highest in *Bufotes variabilis* (64%) followed by *E*. *calamita* (40%), *Bombina bombina* (34%) and *R*. *arvalis* (13%) (Table 2). The prevalence was very low in *Bufo bufo* and *R*. *temporaria* (just five of 341 and one of 197 individuals of these species, 1.5 and 0.5%, respectively, were infected; Tables 2 and S2), so they were excluded from both the pond- and landscape-level analyses.

**Table 2:** Information on species sampled and screened for *Bd* infection in southern Sweden.

Species	Sampling 2015 Day/Mo	Sampling 2016 Day/Mo	Bd+/No. sampled (Prevalence, %)	Prevalence 95% CI
*Bombina bombina*	08-17/5	-	10/29 (34.5)	18–54
*Bufo bufo*	27/3-11/4	04-17/4	5/341 (1.5)	0.5–3.3
*Bufotes variabilis*	01-21/5	02-09/5	86/134 (64.2)	55–72
*Epidalea calamita*	01-11/5	10/5	33/83 (39.8)	29–51
*Rana arvalis*	27/3-14/4	02-12/4	21/163 (12.9)	8–19
*Rana temporaria*	25/3-11/4	07-17/4	1/197 (0.5)	0–3.0

Numbers of individuals and sampling periods for each species in 2015 and 2016 (Sampling 2015 and Sampling 2016), including detected *Bd*-positives (Bd+), total number of sampled individuals (No. sampled) and detected prevalence in percentages.

**Fig 2:**
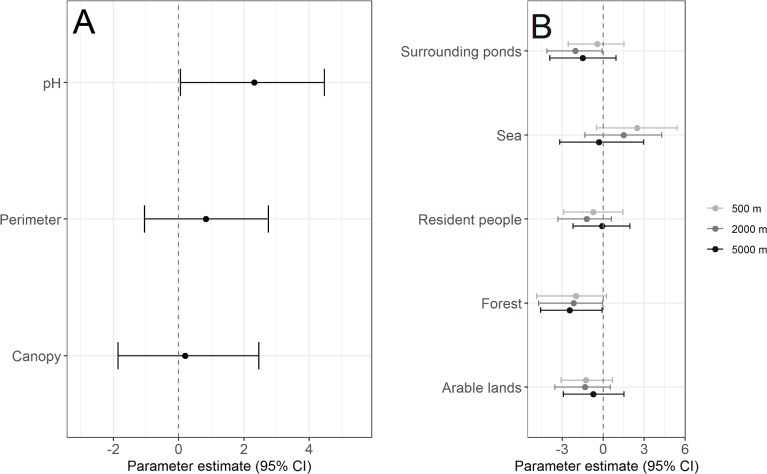
Associationsbetween detected *Bd* infection prevalence and local/landscape factors. (A) local environmental factors (pond characteristics) and (B) landscape environmental factors. In (B) different coloured points/bars represent prevalence estimates at the indicated spatial scales. Points represent posterior modes and error bars 95% Bayesian credible intervals of *Bd* infection prevalence. Deviations of bars from zero represent significant differences. Results including species (fixed effects) and random effects are presented in S3 Table.

**Fig 3:**
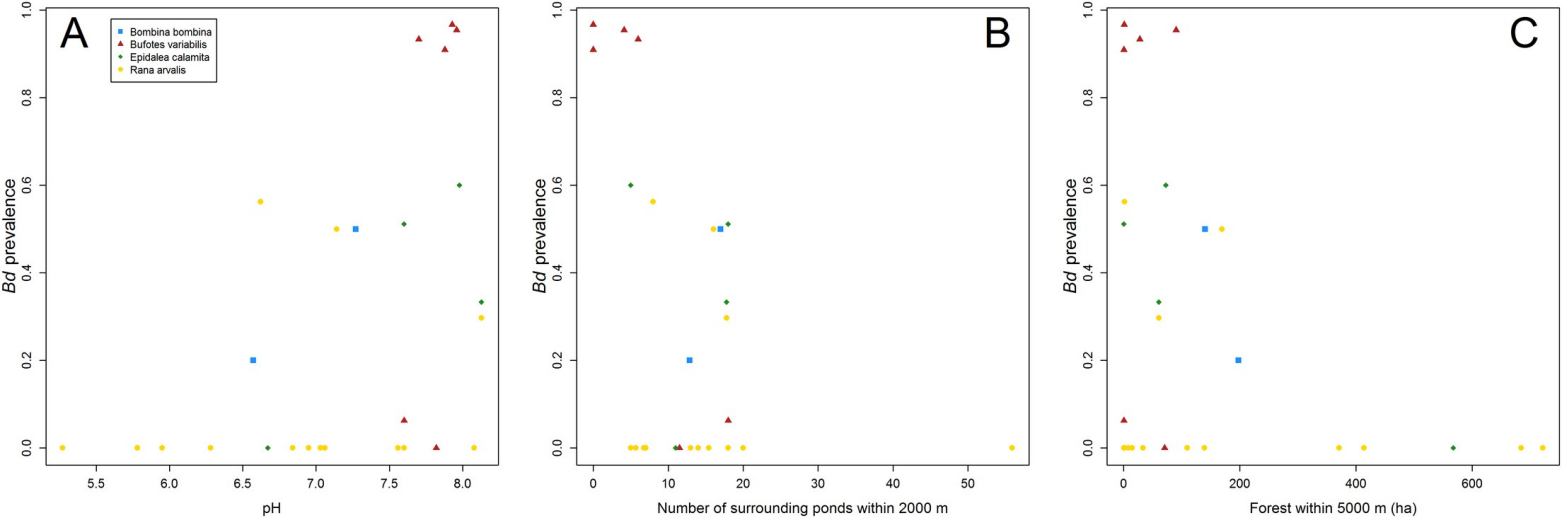
Mean estimates per site and species of detected *Bd* prevalence plotted against three important predictors. (A) Pond pH, (B) number of surrounding ponds within 2000 m, and (C) hectares of mature forests within 5000 m.

One of the pond variables, pH, was positively associated with the detected *Bd* infection prevalence (Figs 2A and 3A), while Perimeter and Canopy had at most weak associations. *Surrounding ponds* in the landscape were negatively associated with detected *Bd* infection prevalence at the 2000 m spatial scale (Fig 2B) and six of the ponds with the highest infection prevalence (>50%) had less than ten surrounding ponds (Fig 3B). Mature forest was negatively associated with detected *Bd* infection prevalence at the two largest scales (Fig 2B). All ponds that yielded *Bd-*positive samples had less than 200 ha mature forest within 5000 m (Fig 3C). *Bd* infection patterns in *Bufotes variabilis* and *E*. *calamita* were the main contributors to the associations of *Bd* infection prevalence with pH, Mature forest and Surrounding ponds.

Based on the weighting of the MCMC models, the local pond factors explained more variation in *Bd* infection prevalence than the landscape factors. The landscape environmental factors within a 5000-m radius explained more variation than the factors within 500 and 2000 m radii (Table 3).

**Table 3:** Variation in *Bd* infections explained by the models.

Model	DIC	Model weight	BIC	Model weight
Local factors	278.7	0.392	287.6	0.997
Landscape, 500 m	280.6	0.153	301.0	0.001
Landscape, 2000 m	281.2	0.116	302.2	0.001
Landscape, 5000 m	279.0	0.340	300.6	0.002

DIC (Deviance Information Criterion), BIC (Bayesian Information Criterion) and model weights of local (pond characteristics) and landscape factors generated from the Markov-chain Monte Carlo simulation-based models. Variables used in each of the models are presented in Table 1.

Ponds sampled for *Bd*-infected anurans were widely spread across the study area (Fig 1), and *Bd* infection prevalence was not spatially correlated when data for all species were included (Moran’s I = -0.058, p = 0.192), or for the four species included in the local and landscape models (Moran’s I = -0.059, p = 0.627). No significant differences in detected prevalence of *Bd* infections were found between years when data for all species were included (2015 = 0.168, 2016 = 0.178, χ^2^ = 0.00, *p* = 1.00). However, a significant between-year difference was found for the four species included in the local and landscape models was detected (2015 = 0.283, 2016 = 0.429, χ^2^ = 4.0338, *p* = 0.04).

## Discussion

Understanding associations between *Bd* infection dynamics, environmental factors and amphibian diversity is important for mitigating adverse effects of the chytrid on amphibian populations. However, there is little information on these associations in high temperate latitudes, therefore we have examined associations between *Bd* infection prevalence and both local factors and landscape metrics in southern Sweden. We found that the amphibian host species as well as breeding habitat characteristics and landscape factors were associated with the prevalence of *Bd* infection. Our results highlight the potentially complex interplay between pathogen occurrence patterns, local habitat conditions, and landscape characteristics.

### Species differences

Species differed in *Bd* infection prevalence, with *Bufo bufo* and *R*. *temporaria* showing the lowest prevalence. Temperatures in which these two species have been sampled, during their breeding seasons, are commonly below 10°C ([73–74] S. Kärvemo, unpublished data). Thus, their low *Bd* infection prevalence may be at least partly due to low temperatures during these species’ breeding seasons, as temperature is reportedly correlated with *Bd* growth rates [51, 75]. The other four anuran species sampled at our study sites breed at higher temperatures, from a few days to several weeks later than *Bufo bufo* and *R*. *temporaria*, which may contribute to their higher *Bd* infection prevalence. A potential temperature effect is supported by the findings that *Bd* prevalence was higher in 2016 (May mean temperature: 13.6°C) than in 2015 (May mean temperature: 9.9°C), when mean air temperatures were also higher, according to data from a weather station in Malmö (Swedish Meteorological and Hydrological Institute). Alternatively, *Bufo bufo* and *R*. *temporaria* may have better immune defence against *Bd* in terms of, for example, major histocompatibility complex (MHC), antimicrobial peptide (AMP) variants coding for resistance alleles [76–77], or skin microbiota with antifungal properties [29]. The species with the highest detected *Bd* infection prevalence were *Bufotes variabilis* and *E*. *calamita* and their infection patterns seem to have been the main contributors to the associations between *Bd* infection prevalence and environmental factors. As these species generally inhabit specific environments, it is difficult to distinguish if the environmental factors are directly linked to *Bd* or the results reflect the habitat choices of susceptible amphibian species.

### Local factors

Pond pH was strongly positively associated with *Bd* infection prevalence, particularly when pH was higher than 6.5 (Fig 3A). This is consistent with observations of increases in *Bd* growth rates with increases in pH in previous experimental [51] and field studies [78–79]. It is not clear why *Bd* infection is affected negatively by low pH. However, Chestnut et al. [79] suggested that it may be due to reductions in metabolic rates of the fungus and organic carbon substrates, which are important nutrients for aquatic fungi in low pH environments.

Several variables affecting water temperatures have been reported associated with prevalence of *Bd* infection, for example, local canopy cover [4, 36] and elevation [24, 42]. Canopy cover was not a predictor of *Bd* prevalence in our dataset, probably because canopy cover did not exceed 65% at any of the sites and only exceeded 50% at three sites, which may have resulted in too little variation to detect effects. Similarly, the variation in elevation of our study sites was very limited (range, 37.5–170.9 m a.s.l.) and this variable was not included in the analyses after preliminary screening.

According to DIC (Deviance Information Criterion) analyses, local factors explained most of the detected variation in *Bd* infection prevalence, or similar amounts to the landscape factors at the largest scale. However, BIC (Bayes Information Criterion) analyses, which impose penalties for increases in the number of factors in a model, clearly indicate that local factors explained more of the variation. Therefore, our results support the role of local factors included in the models being somewhat more important than the landscape factors.

### Landscape factors

The landscape metric most consistently associated with *Bd* infection prevalence across spatial scales was Mature forest, particularly at the two largest scales. In contrast to our results, earlier studies at various spatial scales have reported that forests were associated positively with occurrence of *Bd* [31, 37, 42]. However, the cited studies focused on sites in regions with warmer climates, e.g. California [42] and Romania [37], where the mean spring temperatures are 3–8°C warmer than in southern Sweden (www.weatherbase.com). Accordingly, the reason for the negative association of forests with *Bd* infection at our study sites could be that the forested environments are too cool and avoided by potential amphibian host vectors [80]. This may be especially applicable to the four species included in our analyses, as at least three of them are considered to be adapted to warm environments and prefer open habitats [81].

The number of surrounding ponds was negatively associated with *Bd* infection prevalence at the 2000-m scale, within the range of common migration distances for pond-breeding amphibians [63]. We hypothesize that the detected negative correlation may be due to a dilution effect, induced by increases in amphibian biodiversity and density with connectivity, which may, in turn, decrease the prevalence and infection intensity of *Bd* [82, 83]. Although the differences between the scales were small, the landscape variables at the 5000-m scale explained most of the variation in *Bd* infection prevalence. This is within a feasible dispersal distance for anurans [63] and suggests interactions of environmental factors and *Bd* at this broader scale.

## Conclusions

Our results suggest that the importance of environmental factors for *Bd* infection prevalence varies across species and spatial scales. For future investigations, it is therefore highly important to choose relevant scales and avoid drawing general conclusions based on observations of a single host species. In general, our findings indicate that local factors have more influence than landscape factors on *Bd* infection prevalence, but landscape factors may strongly influence amphibian distribution patterns and hence *Bd* infection patterns. The results from this study may be useful for formulating amphibian conservation measures in northern Europe by mapping species, ponds and landscapes with high risk of *Bd* infections. They may also assist efforts to counter further spread of the disease and develop species-specific conservation action plans, especially for the most threatened species in northern Europe.

It is difficult to distinguish if the environmental factors are directly linked to *Bd* or the results reflect habitat choices of susceptible amphibian species. Nevertheless, the results indicate that in cooler climates the associations between landscape factors and *Bd* infection prevalence may differ from those at lower latitudes or altitudes. Specifically, amphibians inhabiting ponds with relatively high pH may face a higher risk of *Bd* infections, whereas individuals inhabiting landscapes with many surrounding ponds and large areas of mature forests may have a reduced risk. Importantly, many of the environmental factors considered in this study are modified by humans over time. Without thorough cross-scale analyses, researchers and stakeholders may overlook the impact of human-mediated changes in the landscape on amphibians, ecosystems and overall biodiversity [72].

## Supporting information

S1 Table Summary table over data: Including detected presence of chytrid (*Bd*), number of infected individuals (Bd+), number of non-positive individuals (Bd-), *Bd* infection prevalence per site including all species (prev all), species sampled 2015 (Species 2015) and 2016 (Species 2016): Bb = *Bufo bufo*, Bob = *Bombina bombina*, Bv = *Bufotes variabilis*, Ec = *Epidalea calamita*, Ra = *Rana arvalis*, Rt = *Rana temporaria*, prevalence of the four species (bold) used in the main models (prev 2015 and prev 2016), prevalence used in the local and landscape models (prev model), pH is from the sites sampled in 2017 (pH), perimeter of the ponds (Perimeter (m)), percentage of southern canopy covering the ponds recorded 2017 (Canopy (%)), hectare of mature forest in the landscape (Forest landscape (ha)), hectare of arable land in the landscape (Arable landscape (ha)), number of surrounding ponds in the landscape (No.of ponds landscape), number of resident people in the landscape (No. people landscape), and the presence of sea (Sea landscape). Bold information denotes data included in the local and landscape models. Light gray is the variables used in the local factor model and dark gray is the variables used in the three landscape models.
(XLSX)

S2 Table Six species and differences in detected *Bd* infection prevalence. Bayesian mixed-effects models of *Bd*-prevalence and the six species of amphibians with prevalence in *Bombina bombina* as the intercept against which prevalence in the other species was tested. Mean (post.mean), lower (l-95% CI) and upper (u-95% CI) 95% confidence intervals, effective sample sizes (eff.samp) and p-values (pMCMC).
(DOCX)

S3 Table Results from the local and landscape models at three spatial scales and associations with detected *Bd* infection prevalence in the four-species Bayesian mixed-effects models. Mean (post.mean), lower (l-95% CI) and upper (u-95% CI) 95% confidence intervals, effective sample sizes (eff.samp) and p-values (pMCMC). Prevalence in *Bombina bombina* is the intercept against which prevalence in the other species was tested. Bold p-values denote significant results.
(DOCX)
