# Supplementary material for: Effects of host species and environmental factors on the prevalence of Batrachochytrium dendrobatidis in northern Europe
Source: PLoS One. 2018 Oct 25;13(10):e0199852. doi: 10.1371/journal.pone.0199852 (PMC6201871; doi:10.1371/journal.pone.0199852)
Supplement: S2 Table — Bayesian mixed-effects models of Bd-prevalence and the six species of amphibians with prevalence in Bombina bombina as the intercept against which prevalence in the other species was tested. Mean (post.mean), lower (l-95% CI) and upper (u-95% CI) 95% confidence intervals, effective sample sizes (eff.samp) and p-values (pMCMC). (DOCX) [file pone.0199852.s002.docx]

**S2 Table**

|  | post.mean | l-95% CI | u-95% CI | eff.samp | pMCMC |
| --- | --- | --- | --- | --- | --- |
| Intercept (*B. bombina*) | -2.87 | -4.84 | -0.94 | 1000 | - |
| *Bufo bufo* | -3.78 | -5.82 | -1.96 | 1000 | **<0.001** |
| *Bufotes variabilis* | 1.10 | -0.90 | 2.86 | 1000 | 0.232 |
| *Epidalea calamita* | 2.10 | 0.16 | 3.84 | 1000 | **0.016** |
| *Rana arvalis* | 0.15 | -1.46 | 2.33 | 1000 | 0.920 |
| *Rana temporaria* | -3.90 | -6.04 | -1.72 | 1000 | **<0.001** |
