# Supplementary material for: Effects of host species and environmental factors on the prevalence of Batrachochytrium dendrobatidis in northern Europe
Source: PLoS One. 2018 Oct 25;13(10):e0199852. doi: 10.1371/journal.pone.0199852 (PMC6201871; doi:10.1371/journal.pone.0199852)
Supplement: S3 Table — Mean (post.mean), lower (l-95% CI) and upper (u-95% CI) 95% confidence intervals, effective sample sizes (eff.samp) and p-values (pMCMC). Prevalence in Bombina bombina is the intercept against which prevalence in the other species was tested. Bold p-values denote significant results. (DOCX) [file pone.0199852.s003.docx]

**S3 Table**

| Local factors | post.mean | l-95% CI | u-95% CI | eff.samp | pMCMC |
| --- | --- | --- | --- | --- | --- |
| **Fixed effects** |  |  |  |  |  |
| Intercept (*B. bombina)* | -1.347 | -3.806 | 1.380 | 1000 | 0.286 |
| *Bufotes variabilis* | -2.089 | -4.324 | 0.351 | 1000 | 0.086 |
| *Epidalea calamita* | 0.355 | -1.788 | 2.459 | 1000 | 0.768 |
| *Rana arvalis* | -0.512 | -2.830 | 1.659 | 1000 | 0.680 |
| Perimeter | 0.764 | -1.138 | 2.791 | 1000 | 0.406 |
| pH | 2.386 | 0.171 | 4.574 | 1000 | **0.034** |
| Canopy | 0.184 | -1.826 | 2.163 | 863.3 | 0.818 |
| **Random effect** |  |  |  |  |  |
| Site | 28.650 | 6.043 | 62.100 | 1000 |  |
| Landscape factors, 500 m |  |  |  |  |  |
| **Fixed effects** |  |  |  |  |  |
| Intercept (*B. bombina*) | -2.232 | -4.727 | 0.332 | 1000 | 0.098 |
| *Bufotes variabilis* | -2.250 | -4.466 | -0.054 | 1000 | **0.038** |
| *Epidalea calamita* | 0.133 | -1.898 | 2.275 | 1000 | 0.892 |
| *Rana arvalis* | -0.987 | -3.170 | 1.113 | 1000 | 0.384 |
| Surrounding ponds | -0.385 | -2.482 | 1.683 | 1000 | 0.702 |
| Forest | -1.944 | -4.587 | 0.549 | 872.4 | 0.102 |
| Arable land | -1.204 | -3.051 | 0.411 | 511.5 | 0.152 |
| No. of resident people | -0.693 | -2.920 | 1.425 | 1000 | 0.524 |
| Sea | 2.558 | -0.568 | 5.313 | 1000 | 0.104 |
| **Random effect** |  |  |  |  |  |
| Site | 19.550 | 3.170 | 46.750 | 897 |  |
| Landscape factors, 2000 m |  |  |  |  |  |
| **Fixed effects** |  |  |  |  |  |
| Intercept (*B. bombina*) | -1.927 | -4.565 | 0.549 | 1450 | 0.142 |
| *Bufotes variabilis* | -2.238 | -4.595 | -0.181 | 1277 | 0.052 |
| *Epidalea calamita* | 0.114 | -2.106 | 2.116 | 1000 | 0.922 |
| *Rana arvalis* | -0.833 | -2.953 | 1.368 | 1000 | 0.442 |
| Surrounding ponds | -2.108 | -4.233 | 0.051 | 1000 | **0.046** |
| Forest | -2.041 | -4.981 | -0.153 | 1000 | 0.056 |
| Arable land | -1.289 | -3.389 | 0.546 | 894 | 0.212 |
| No. of resident people | -1.306 | -3.429 | 0.890 | 1000 | 0.186 |
| Sea | 1.516 | -1.284 | 4.330 | 1000 | 0.298 |
| **Random effect** |  |  |  |  |  |
| Site | 15.400 | 2.380 | 38.380 | 943 |  |
| Landscape factors, 5000 m |  |  |  |  |  |
| **Fixed effects** |  |  |  |  |  |
| Intercept (*B. bombina*) | -1.234 | -4.350 | 1.280 | 1000 | 0.384 |
| *Bufotes variabilis* | -2.059 | -4.312 | 0.347 | 1000 | 0.076 |
| *Epidalea calamita* | 0.508 | -1.541 | 2.805 | 1286 | 0.652 |
| *Rana arvalis* | -0.456 | -2.553 | 1.728 | 1000 | 0.702 |
| Surrounding ponds | -1.581 | -3.988 | 0.520 | 1000 | 0.150 |
| Forest | -2.436 | -4.575 | -0.449 | 1353 | **0.010** |
| Arable land | -0.580 | -3.103 | 1.786 | 1000 | 0.640 |
| No. of resident people | -0.013 | -2.099 | 2.168 | 1095 | 1.000 |
| Sea | -0.287 | -3.368 | 3.120 | 1000 | 0.870 |
| **Random effect** |  |  |  |  |  |
| Site | 24.940 | 5.739 | 57.060 | 664 |  |
